# Supporting emergency service workers to cope with critical incidents that can lead to psychological burden at work - developing skills in the Post Critical Incident Seminar: a qualitative interview study

**DOI:** 10.1186/s40359-024-01534-x

**Published:** 2024-01-22

**Authors:** Sanna Korpela, Hilla Nordquist

**Affiliations:** South-Eastern Finland University of Applied Sciences, Pääskysentie 1, Kotka, 48220 Finland

**Keywords:** Post-critical incident seminar, Firefighter, Paramedic, Psychological burden, Coping

## Abstract

**Background:**

Emergency service workers face critical incidents causing psychological burden. This qualitative study investigates how attending a Post Critical Incident Seminar could affect the skills of emergency service workers to overcome incidents that can cause psychological burden at work and their consequences with the following research questions: (1) How did attending the Post Critical Incident Seminar impact the skills to overcome work incidents that can lead to psychological burden? and (2) How have these skills been manifested since attending the Post Critical Incident Seminar?

**Methods:**

The data consists of individual interviews with fifteen emergency services workers who attended a Post Critical Incident Seminar in April 2021 in Finland. The interviews were conducted six months after the Post Critical Incident Seminar and analysed using inductive content analysis.

**Results:**

The results of the first research question formed two main categories: improved performance abilities and improved self-regulation abilities. From the results of the second research question two main categories were created: new kind(s) of well-being and readiness to help. Several upper categories and sub-categories were discovered.

**Conclusions:**

Based on the results, attending a Post Critical Incident Seminar may be effective in developing skills to overcome critical incidents that can lead to psychological burden. Further follow-up studies should investigate how acquired skills from the Post Critical Incident Seminar practically affect emergency service workers and their well-being in the longer term.

**Supplementary Information:**

The online version contains supplementary material available at 10.1186/s40359-024-01534-x.

## Introduction

There is a high probability that emergency service workers, such as firefighters and paramedics, face critical incidents that can result in psychological burden. In this study, we use the term psychological burden when referring to negative psychological influence on a person over a longer period, not only momentarily, but also typically comes from encountering demanding situations. It can be viewed as a negative psychological consequence of a single intense experience or multiple experiences, but it may also stem from a prolonged period of heavy workload [1–3]. The degree to which this poses a problem depends on the situation, the organisation, and the individual factors as to how these incidents may affect the person. However, the frequency of these incidents increases the probability of psychological burden and other mental health symptoms experienced by emergency service workers [4–6]. 

Compared to other workers, emergency service workers have a higher risk of Post-Traumatic Stress Disorder (PTSD) [7]. This also applies when compared to other populations; for example, the prevalence of PTSD among paramedics is considerably higher than in the general population [8]. The risk is not only related to PTSD. In addition, the sense of coherence, which refers to a person’s internal resources to cope with stressful situations, has been linked to predicting PTSD severity in volunteer firefighters [9]. Moreover, it has been noted that among paramedics, there can be a lower overall psychological, physical, and social well-being [1, 10].

Since there is an increased risk for mental health issues and psychological burden among emergency service workers, there is a clear need to identify what kind of coping skills and support mechanisms are used and how these could be developed [1, 3, 11]. Regardless of exposure to incidents resulting in psychological burden, emergency service workers rarely seek professional help [2]. For example, in a study researching firefighters involved in the response at the World Trade Centre in the United States on September 11, 2001, the relationship between perceived job control and delay in seeking help was not straightforward. Moreover, the highest levels of delay in seeking help have been found to exist when the perceived job control is very low or very high under conditions of more extreme situational severity [12].

In this study, we are interested in the skills of emergency service workers to cope with incidents that cause psychological burden at work. Defining skills, as well as their limits, as concepts is not a straightforward process. In this study, we have defined skills to include actions and behaviours that can be seen as acts in practice and ways of thinking that refer more to internal cognitive or emotional processes, such as implementation and regulation capabilities at work and outside of work.

It has been noted that instead of seeking professional help, emergency service workers may try to help themselves in other ways, mostly through maladaptive methods, such as avoiding recurring feelings and thoughts about the incidents they faced, which leads to psychological burden and the development of mental disorders [13]. Moreover, drinking has been found to be a way of coping after experiencing an incident that cause psychological burden [14], but it is associated with poor performance and other alcohol-related health concerns [15]. Additionally, for example, the level of resources at the work unit level, meaning support, tools, and strategies available in a workplace, can affect the psychological responses to experienced incidents and drinking to cope [14]. According to a recent systematic review [13], adaptive, active coping strategies could be protective for emergency service workers, including acceptance, positive reinterpretation, focusing on the problem, self-efficacy, and social or instrumental emotional support. However, these are not sufficiently utilised, so the skills needed to utilise these strategies should be strengthened [13]. 

In a study of Greek firefighters, it was found that among different types of coping mechanisms, minimisation (regarding the importance of the incident that can lead to psychological burden) and blame are mostly associated with PTSD [16]. Dysfunctional coping style, more specifically avoidant coping mechanism, is connected with post-traumatic stress symptoms [17]. However, in a Canadian study, it was found that public safety personnel, including emergency service workers with or without mental health diagnosis, reported lower intolerance for uncertainty and anxiety sensitivity than individuals with diagnosed mental disorders representing the general population [18]. Thus, more understanding of functional coping skills is needed when facing critical incidents that can lead to psychological burden.

There have been some support mechanisms offered for emergency service workers in Finland to cope with critical work incidents. One of them is defusing. In Finnish emergency services, defusing means a discussion session led by a trained peer after a heavy work task or incident. It is not as intense or as strictly structured as debriefing. Different geographical areas in Finland have different guidelines. Further, support from occupational health services, such as occupational health psychologists, is available.

Since 1986, the Post Critical Incident Seminar (PCIS) is a support intervention where participants are provided psychoeducation, peer support and professional services including Eye Movement Desensitization and Reprocessing (EMDR) that was originally developed to help police officers of FBI (Federal Bureau of Investigation) who have experienced an incident causing psychological burden at work [19]. Although it has been used for many years, there are not many studies about PCIS and its effectiveness on the well-being of the participants [20–22]. One study that compared PTSD, anxiety, and depression symptoms of police officers before and after attending PCIS discovered a significant change in all these measures was achieved [21]. Another study [22] reported that PTSD symptom scores significantly decreased six months after participating in PCIS.

There is a clearly identified need to understand better the long-term effectiveness of skills to cope with incidents that can lead to the psychological burden of emergency service workers [23]. It is evident that enhancing the skills to recover from critical incidents is important for the individual’s well-being and ability to conduct their work successfully. Furthermore, it is important from the system perspective that the workforce resources are functional to handle and recover from larger and smaller incidents as an entity [24]. When workers can cope with incidents leading to psychological burden of different scales, they can also mitigate the negative consequences for the community, system and society.

In this study, we investigated how attending a Post Critical Incident Seminar (PCIS) could affect the skills of emergency service workers to overcome incidents that cause psychological burden at work and their consequences. Accordingly, the research questions were: (1) How did attending a PCIS impact the skills to overcome work incidents that can lead to psychological burden? And (2) How have these skills been manifested since attending the PCIS?

## Materials and methods

This qualitative interview study is part of a larger Finnish research project which aims to investigate the effectiveness of the Post Critical Incident Seminar.

### Emergency service workers in Finland

At the time of this study (2021), Finland had 21 rescue departments managed by municipalities responsible for rescue service duties in their regions. Since the beginning of 2023, rescue departments have been part of well-being service counties, except for the City of Helsinki. Before 2023, the responsibility for organising prehospital emergency medical services lay with hospital districts, and rescue departments could also provide these services if it was locally agreed upon. Since the 2023 reform, the well-being services counties have been responsible for organising prehospital emergency medical services. As the same well-being services county now organises the rescue services, the rescue department can also provide emergency medical services. The 2021 PCIS was targeted to emergency service workers (meaning firefighters, paramedics, and firefighter-paramedics) employed by the rescue departments.

The Finnish firefighter qualification is a 1.5-year vocational training that can be completed at the Emergency Services Academy Finland or the Helsinki Rescue School. Graduates also have the qualifications to work as firefighter-paramedics (basic-level paramedics). The training paths for paramedics are more diverse. Basic-level paramedics are registered nurses without a prehospital specialisation (bachelor’s degree in nursing = 3.5 years training at a University of Applied Sciences) or practical nurses with a prehospital specialisation (3 years vocational training). To work at the advanced level, the paramedic must have a bachelor’s degree in emergency care (4 years of training in a University of Applied Sciences) or a bachelor’s degree in nursing with additional advanced-level prehospital specialisation.

The Finnish Association of Fire Officers (established in 1932) has a central goal of promoting societal safety and developing the professional expertise of its members but does not engage in professional advocacy activities. The membership includes, among others, fire officers in the emergency service field, contract fire officers, safety experts, and individuals in leading positions.

### The Post Critical Incident Seminar

This emergency service personnel’s PCIS was organised by the Finnish Association of Fire Officers in April 2021. The participants (*n* = 16) of the seminar were the target group of this study. The participants were chosen for the PCIS via an application process by the seminar organisers. According to the organisers, the communication regarding the seminar application clearly conveyed to interested individuals the types of topics that would be processed. In their applications, applicants described the potential incidents they wished to discuss. Incidents were assumed to be from work and influencing their life in general or from private life and influencing their work in emergency services. Specific incidents were not required as it was also possible that a participant had a cumulative psychological burden.

The authors of this study had no role in the application or selection process. The manager and the leading psychotherapist of this particular PCIS conducted the selection. The participants were selected based on their psychological need, willingness, ability and readiness to process their experiences, as described in their written applications. Additionally, how they could benefit from PCIS and how the group dynamics would evolve were considered. The exclusion criteria included if the applicant needed more supervision than PCIS, the incident had happened approximately less than six months ago, or ongoing psychotherapy was already taking place (not to interfere with that process).

The organisers of PCIS defined a six-month waiting period based on the idea that natural healing may still take place, and at that time, it can be seen what kind of symptoms and struggles are still left. The organisers assumed that attending PCIS too soon may interfere with natural healing. The time frame was not strictly kept at six months but was based on consideration of individual cases. Further, if there were physical injuries, they should be taken care of before attending PCIS. The manager arranged at least one supportive discussion with each participant before the PCIS. The supportive discussion before PCIS was organised to lower the threshold of attending and slightly construe their experiences.

The three-day PCIS followed the model used by the FBI [19] with some modifications by the organisers, considering the financial aspects in terms of content and duration (fewer participants, shorter time). The PCIS consisted of psychoeducation and other lectures, peer support (including sharing their own stories to join PCIS and listening to the instructors’ stories), and support discussions with a specialist (psychotherapist with EMDR and possibly Eye Movement Desensitization EMD competence, though this was not considered health care by the PCIS organisers). The seminar program is provided in Appendix 1. This PCIS was the second time a seminar of its kind had been arranged for emergency service workers in Finland. Everyone who participated in the seminar was provided with the opportunity to participate in the research project, including this study.

### Participants

The PCIS participants (*n* = 16) were emergency service workers, meaning firefighters, paramedics, and firefighter-paramedics. They were men and women who worked at different fire departments in Finland. All the PCIS participants had experienced one or several critical incidents at work or in private life, or they had cumulative experience of a psychological burden.

The PCIS participants were informed verbally and in writing about the research project by the last author (female), a senior researcher (PhD), who visited the PCIS in person on the first morning. All PCIS participants provided their informed consent to participate in the research project. The authors of this study did not participate in the seminar days in any other way. Six months after the PCIS, the last author sent a short message to the participants and introduced the interviewer (first author, female) as they had not met her before, intending to establish trust. The message explained that although the interviewer (first author) is a psychologist (MA), in this context, she was a researcher, not a psychologist conducting work with clients. In addition, the interviewer’s professional experience and interest in the research topic and trauma in general were broadly described. After this introduction, the first author contacted the participants and scheduled an individual interview time with them. All of the participants were still willing to participate in the study.

### Conducting the interviews

The interview guide was developed for the research project and is provided in Appendix 2. The semi-structured interview themes included: (1) background information (age, work experience years in emergency services, and time passed since the possible incident), (2) the possible impacts of the PCIS on the experience of the possible critical incident, different areas of life, and psychological state, (3) support or treatment received and possible gaps, before and after the PCIS for reducing psychological burden, and (4) impacts and manifestation of the PCIS on the competencies and skills needed to cope with work incidents that can lead to psychological burden. Each interview theme contained several pre-planned questions, and based on the answers, additional questions were asked during the interviews as the conversation flowed.

Prior to the actual interviews, the first author conducted a test interview with an external person who represented the target group. Feedback from the interviewee improved the interviewer’s understanding of how to conduct the interviews in the most feasible way.

Prior to the actual interviews, the first author contacted every participant (*n* = 16) again and asked them to provide written consent for attending the interview. In addition, the semi-structured interview themes (overall thematic headlines) were sent to the participants prior to the interviews. Thematic headlines were sent to the participants before the interview (together with the consent form) to allow the participants to orientate to the interview, which can also be emotionally heavy due to the discussion of very personal or difficult issues.

After the third interview, the authors decided to change the order of the original third and fourth themes based on the first author’s understanding of how the questions were experienced and answered. Thus, the theme originally as a third became forth.

The interviews were primarily arranged online (Teams), except one conducted face-to-face. The interviews were recorded with two voice recorders, and no video was recorded. One participant did not provide written consent and did not arrive online at the mutually agreed-upon time. Thus, the final number of participants was 15 (94%).

The overall duration of the interviews was 1,014 min. The average length of an interview was 63 min (shortest 52 min, longest 98 min). One interview had to be conducted in two parts (approximately 61 min and 19 min) due to issues with the recorders’ battery. The voice recordings were transcribed by an external company committed to the data protection policy. The transcriptions comprised 321 pages (font size 8, font style Verdana, line spacing 1.08). The transcript data covering the fourth theme is altogether 20 460 words. The whole transcript data covers 120 295 words (excluding background information 1 990 words). The transcript was exact and thus, it included everything that was said and audibly expressed.

### Data analysis

The interview material was analysed with inductive content analysis [25]. Only the parts of the data that were under the fourth theme (according to the original order) were included in this analysis. After generally familiarising herself with the data, the first author coded the interview content (meaning participants’ words and sentences) that answered the research question. This was done using open coding, where headings were collected in the document margins. After this, she copied the headings to a separate document, printed the document, and manually grouped the headings with similar content, forming sub-categories. Then, the sub-categories were again grouped under upper categories. Finally, these categories were grouped under the main categories that generally describe the research question. Throughout this process, the first and the last authors discussed the analysis and its results, aiming to maximise the quality of the analysis.

## Results

Fifteen emergency service workers participated in this study. The participants were over 31 years of age, and most of them had more than 11 years of work experience in the emergency services (Table 1).

**Table 1:** Age of the participants and work experience in emergency services

	*n* = 15
**Age (years)**	
31–40	5
41–50	6
51–60	4
**Work experience in emergency services (years)**	
0–5	0
6–10	2
11–15	10
16–20	1
20+	2

### The impact of PCIS on the skills needed to overcome incidents that can lead to psychological burden

The results regarding the research question “How did attending a PCIS impact the skills to overcome work incidents that can lead to psychological burden?” formed two main categories. These were grouped into five upper categories and 18 sub-categories. The main categories, upper and sub-categories, are presented in Table 2.

**Table 2:** The main, upper and sub-categories of research question 1

Main categories	Upper categories	Sub-categories
Improved performance abilities	Improved action preparedness and ability	Readiness for better performance Better readiness for working and facing clients Personal resources better utilised Better understanding of the psyche and its symptoms
	Ability to better help themselves	Skills to regulate own state of mind Understanding how to help themselves (during work tasks) Improved tools Readiness to use organisational peer support mechanisms more
Improved self-regulation abilities	Revived approach to self and things	Seeing things from a new angle Relieved presence (in this moment) Relieved approach to previous incidents
	Improved interaction-based skills	Finding support network Perceiving examples Openness to discuss Listening to others
	Learning to accept own needs and emotions	Mercifulness towards themselves Facing emotions and normalising them Listening to themselves and recognising needs

#### Improved performance abilities

##### Improved action preparedness and ability

The participants mentioned having significantly better stress management skills, the ability to vent tasks that cause burden, and the feeling a return of their ability and trust in making decisions. Further, participants mentioned the ability to ignore things that they cannot have control over and could recognise situations where they need to calm themselves down, and trusting that they are able to do it that increases their performance level and trust in it.

The participants described sensitivity to listening without becoming anxious themselves but being able to help and being more alert when meeting clients. They also mentioned having the psychological resources to work professionally and being brave at work.

*“Probably such as sensitivity, what I mentioned already that when one is more sensitive to listen, listen to others then, and not getting anxious themselves but I am able to sort of well, to help also the other person by listening and well, by sympathising with their problems then better. And perhaps even to bring some sort of thought about it, well, how to go forward from this. That it is probably the sort of well, the big-biggest thing.” (Participant 8).*

More and strengthened psychological resources, as well as being able to function within the limits of their own resources, were mentioned.

The participants listed understanding the similarity of bodily reactions in critical incidents or nearly critical incidents and understanding the significance of resting, as well as understand the significance of sleep and restful exercise. Also, they mentioned understanding how the psychological symptoms may be like, and knowledge of what affects psychological well-being. They also described learning to act differently in certain situations due to improved understanding and thinking it is futile to think that the psyche does not affect the way of working.

*“Well in the fire brigade there has long time been that it is just part of the work that you will face bad experiences.… especially in the beginning it was told that if you cannot take it you are in the wrong place. That you should leave if you cannot go through the things… as they have been gone through mainly in sauna and with a bottle. Well then, when there is consciousness that you have gained you learn to understand these things completely differently and do something for them and taking into consideration on your mate too that these situations will be processed.” (Participant 1)*.

*“So perhaps also that it is unnecessary for us to deny that our own psyche would not affect how we work in there” (Participant 9)*.

##### Ability to better help themselves

The participants mentioned having tools to calm themselves down if the pace is too fast, being able to recognise their state of alertness and react to it, as well as calming themselves if they feel threatened. They mentioned being able to lower the level of negative emotions, ignore things that used to cause strong reactions and being able to stay calm easier.

The participants listed understanding how to handle a burdening work task, receiving tools to structure experiences, as well as gaining more understanding towards their own history and reactions. They also described understanding how to protect themselves concretely at work from becoming traumatised and knowing that if something happens, they can overcome it.

*“And to understand totally in a different way it that okay now I had had this type of again this type of hard gig. That that what I need to sort of do now that, how to talk about it and how to handle it, and with whom to handle it and so on. So totally in a different way indeed I understand those things sort of now afterwards. It has indeed been a large effect.” (Participant 1)*.

Gaining tools to look after their well-being was described. Better sleep was also mentioned.

Utilising defusing better, requiring defusing for colleagues, a lower threshold to ask for venting support (assumed referring to defusing), and being ready to talk in defusing situations were mentioned.

#### Improved self-regulation abilities

##### Revived approach to self and things

The participants described the proportions of things had changed, changed way of thinking about life, themselves and emotions, as well as thinking not being so rigid. They mentioned understanding how things should be handled, having more structured and clear thoughts, and distance from difficult emotions. Further, believing they are good without convincing and thinking about things that were accomplished instead of those that were not accomplished were listed.

*“…That not this, not this life is not this way and life can be very different. And of course, that when you have kind of realized afterwards that sort of that what type of a mother you have been and what type of an employee, a colleague you have been so, you realise you were in the deep end…” (Participant 10)*.

The participants described feeling calm, relieved, at ease and being able to concentrate on the present moment. Feeling calm that not everything has to be solved immediately or become ready as a person themselves was mentioned.

The participants mentioned it being easier to handle and understand experiences, and how they were previously not listening to themselves or dealing with their experiences.

##### Improved interaction-based skills

The participants listed using and having support from a network from PCIS later since, with them, there is trust and easiness to talk. It was also described how the things learned at the PCIS are revised when talking with other participants.

Receiving examples from psychotherapists and other participants that have helped them to discuss and understand their situations was mentioned.

*“Well I don’t know I guess naturally that atmosphere of trust was there. That so then you dared there. First you felt that no, I don’t dare to even say such things but then when others said too so then well, let’s jump to the deep end and me too, so I guess that.” (Participant 2)*.

The participants described the importance of being open when receiving help, having trust that discussing will help to go through experiences and understanding that even the little things need to be discussed. Sharing things with others that have helped was described. The participants described becoming more open and talking more about their well-being, being brave in bringing up things that bother them and talking more at home. Having to trust to be able to share their experiences with others was also listed.

The participants mentioned listening better instead of talking (interpreted meaning both colleagues and private life), being more sensitive to listening to others (colleagues) and others’ well-being as well as observing others’ (colleagues) well-being.

##### Learning to accept needs and emotions

Feeling it is allowed to have emotions at work instead of being tough was said. Through peer support, they realised there is nothing to be ashamed of in their experiences and have mercifulness towards themselves and towards resting. They also mentioned that they do not need to try to be perfect anymore and that they previously felt ashamed as they thought they were the only ones to feel that way, and thus, they felt as if they were a worse paramedic than others.

The participants described understanding instead of avoiding anxiety, how facing difficult emotions will become better, understanding how natural and adequate emotions are when facing a critical incident, and being able to anticipate work tasks that may feel difficult. Further, participants mentioned the normalisation of emotions, giving freedom for emotions to be felt without hiding or shame.

Recognising too much of a state of alertness, listening to themselves, and recognising their own needs and limits were mentioned. The participants also described how it was easier to recognise changes in themselves and able to understand their own state of mind and moods.

*“Probably that type of sort of skill, sort of to interpret the own state of mind and… moods about it… so that type of kind of, in a way that not kind of wanting anymore to drift into that type of similar situation, like it was before.” (Participant 6)*.

### Manifesting the gained skills since attending PCIS

The results regarding the research question “How have these skills been manifested since attending PCIS?” formed two main categories, which were grouped from four upper categories, and a further 13 sub-categories. The main categories, upper and sub-categories are presented in Table 3.

**Table 3:** The main, upper and sub-categories of research question 2

Main categories	Upper categories	Sub-categories
New kind(s) of well-being	Burden does not control life anymore	Being able to concentrate on what is wanted New approaches to their burden
	Supporting their own well-being	Supporting their own recovery Protecting themselves with scheduling
Readiness to help	Implementing the learned self-help skills	Still processing Utilising defusing support better Better psychological abilities at work to help themselves Consciously using learned techniques Listening to themselves and consciously regulating their own state of mind
	Helping others and better relationships	Being interested in a helping role Positive change(s) at home Looking after others (colleagues, patients and patients’ family) More open discussions at work

#### New kind(s) of well-being

##### Burden does not control their life anymore

The participants described they feel they do not need to carry others’ worries and can prioritise their own concerns, and they do not care anymore what others think about their struggles with their well-being. Further, they stated that they do other things to forget work, disconnect after work, and concentrate on being present with others. Protecting themselves from burdensome media content in their free time and regulating receiving messages from social media and other forms of digital communications were listed. Further, they mentioned not thinking about others too much when receiving help for themselves and setting limits on what they reveal about themselves at work. The participants mentioned that they ground themselves (It is assumed to refer to self-help exercises that help to bring their attention back to the present moment when the mind drifts elsewhere. This particular participant described sitting on a couch and taking a moment to concentrate on what they preferred.).

*“But exactly that is perhaps it, easiest to ground yourself in a way on that couch. That I take then something else to so that I sort of…forget in a way…” (Participant 4)*.

Surprising themselves and having changed their personality by not dwelling on things as they used to and being able to put aside things that would have previously bothered them for a long time were said. The participants said they can handle issues before they become stressful, understand things they can and cannot control, and accept that, as well as stress less about things than before. It was described how things do not pile up as burdens anymore.

*“…That thing doesn’t have to become as until it is stress when you can handle it already before it.” (Participant 15)*.

##### Supporting their own well-being

The participants mentioned doing recovery activities like yoga, cold water swimming and breathing exercises, calming down and doing pleasant things when feeling very tired as well as resting well so that brains function better. The participants also mentioned deciding to do something other than work, supporting the habit of calmness that helps things run smoothly and not adding burden but decreasing it.

*“… And then of course, it that only simply to decide that now I do something else that that that I spend time for example with loved ones, and it means that then I don’t kind of, then I spend time with loved ones and concentrate on that. And not to necessarily do anything else. And to let also oneself have permission to do that, if sometimes there is time for example to lay on a couch, then it is completely healthy that no need to feel guilty…” (Participant 5)*.

Noticing working too much and burning themselves out and for this reason, taking a break was mentioned. The participants described how they protect their own schedules, take time for themselves, and make choices in everyday life to support their own well-being. The participants also mentioned how their own personal resources are better now due to recognising their needs and limits and understanding how they would be in the same situation as before without taking a break.

*“… well, those own resources stay way better in balance when one recognises those own needs and those limits. So, it increases indeed general energy in even many areas…” (Participant 13)*.

#### Readiness to help

##### Implementing the learned self-help skills

Having continued processing things in therapy was described. They described getting tools and information and recognising too high a state of alertness in PCIS, but they first had to sleep, eat, and exercise or take sick leave before being able to implement change and process things. It was also mentioned how they do not let themselves leave unpleasant things unprocessed anymore.

The participants described using defusing for themselves and colleagues when needed. Further, they said that things are discussed, and defusing support offered more instead of humour.

*“… because it is just as it has been before when there has been sort of joking around about them. So now it is first of all that we talk about them and talk and now we process those things more than before (…) well just that kind of before there was not any defusing particularly so then about these bad gigs… when we talked about them it went easily or it was sort of so called rough humour about them but…” (Participant 7)*.

The participants described paying attention to others’ well-being but mostly focusing on their own well-being. Having an understanding of how to protect themselves at work was said. They also listed that they have ideas at work, and understand and allow emotions that work tasks evoke. Preparing themselves for a critical task and planning how to start was also mentioned.

*“Well even although it doesn’t have to be only as basic resuscitation that if well it is it is indeed indeed in most cases the situation is quite sad then that well the best end result is not not reached that the patient doesn’t stay alive so I can face those loved ones kind of better and help them in that moment, and perhaps to say some comforting word to that to that well what I couldn’t do before and I would have kind of only approximately walked away from there. Now I, I can kind of and dare and have energy to stop to that moment.” (Participant 8)*.

The participants mentioned using calming, grounding, thinking, or breathing exercises when needed at work or home. The participants said they use thinking exercises when they are about to get stuck in their minds or combine that with stress management techniques they have used already before and use calming techniques when travelling with difficult clients.

*“We went through circle of influence there so now many times if it is about to go along to the back bag, so you use the circle to see if you can do anything about it and if I need to carry it with me so sort of, I process things completely in a new way than before. And of course, that processing helps so that I don’t need to put things into the back bag, I mean not necessarily, I have a bit of bad tendency sometimes to swirl about things but well, I dwell very much less, even about the things now.“ (Participant 15).*

Recognising unpleasant feelings and naming them makes pondering the causes possible, listening to themselves, and arranging calming as well as recognising a high state of alertness and calming their actions were mentioned. The participants described that when they recognise that they are feeling down, they can pick themselves slowly up and take it easy to improve their mood. Awareness and processing were described to be happening nearly daily by the participants.

##### Helping others and better relationships

The participants stated being interested in running peer venting sessions (assumed referring to defusing), applying to debriefing facilitator training, and possibly studying to become a therapist.

The participants described the effect of being positive towards their home life, how doing well at home is reflected in everything else and how being open about different emotions at home with family has been useful. Also, being able to tolerate children’s tantrum at home better and being able to soothe themselves if they become upset was said.

The participants described supporting others in seeking help, asking colleagues about their well-being, and supporting them based on their own experience from the PCIS. The participants described recognising and finding it easier to ask about their colleagues’ well-being, thinking previously that it was too sensitive to ask about the well-being of colleagues but seeing it differently now and showing the value of colleagues and their opinions. It was mentioned they are an example of burnout for others if things are going too fast. The participants said they have shared about their lower levels of well-being and encouraging others to take sick leave. The participants mentioned they can support relatives if a patient dies and have the knowledge to face patients suffering from mental health conditions. Also, having more energy to face difficult clients was mentioned.

The participants described how they have discussed psychological burden with their colleagues at work, asked for supervisory support, and raised the importance of handling critical incidents quickly. Coming to talk to the participants more often was described. Taking into consideration psychological issues when training was described. The participants mentioned that sharing emotions openly has helped colleagues, too; emotions are discussed more openly than before, and information is shared. It was described how the culture of discussion had been tried to implement in practice, sharing about PCIS and what kind of good consequences of attending PCIS can cause and sharing more openly about PCIS and one’s own well-being to a manager instead of half-truths.

*“Well, in a way if we think that fire and emergency services well it is thought that there is the role of a hero on their shoulders, and nothing ever feels anywhere. But it is indeed sensed and noticed that indeed the things are felt. So well, so, that way it has maybe also helped kind of the work community too.” (Participant 14)*.

## Discussion

This study addressed two research questions: (1) How did attending PCIS impact the skills to overcome work incidents that can lead to psychological burden? and (2) How have these skills been manifested since attending PCIS? The results of the first research question formed two main categories: improved performance abilities and improved self-regulation abilities. The results of the second research question formed two main categories: new kind(s) of well-being and readiness to help.

PCIS can be seen as a part of the continuum of support for emergency service workers after critical incidents. Compared to other support services, such as defusing or debriefing, PCIS is meant for situations where time has passed since the incident, but symptoms still exist. They are not meant to be compared directly or exclude each other but are more of a complement to each other when needed. These support mechanisms vary in intensity, who provides them, and when. Additionally, they can be divided between preventive or rehabilitating support services.

Overall, psychotherapy, debriefing and critical incident debriefing have shown varied results as rehabilitating interventions [26]. In general, only limited evidence has been shown for post-deployment or post-incident psychosocial support interventions for preventing PTSD or common mental health disorders [11]. In other studies of this research project, the results have also shown promising results [27, 28]. One study revealed positive impacts on both incident-related experiences and psychological state concerning different aspects such as social changes, new perspectives and sensations, incident-related components, future-oriented processes, and new abilities and actions [27]. In another study, it was seen how PCIS can have a decreasing effect on self-reported depression, anxiety and traumatic stress symptoms [28]. PCIS would need to be studied in other study settings to confirm its influence more. PTSD has been seen to be maintained if coping strategies and responses to intrusive memories do not allow emotional processing [29]. PCIS can be seen concentrating on allowing the processing of memories.

### Improved performance abilities

In this study, the upper category of ‘improved action preparedness and ability’ presents aspects describing how to manage different types of situations and issues that are linked to one side of the sense of coherence [9]. Furthermore, the development of coping skills to manage exposure to challenging situations and resilience have been suggested to explain why emergency service workers have low levels of intolerance towards uncertainty and sensitivity towards anxiety [18]. Better stress management skills, the ability to selectively ignore certain things when necessary, and recognizing when self-calming is needed were identified in this study. These factors are linked to another study that highlighted the importance of reducing stress and situational awareness as key contributors to avoiding safety incidents [30]. This study provides detailed insights into the mechanisms behind improved resilience stemming from enhanced performance abilities, which can vary in form. Since being able to perform at the highest possible level is crucial in emergency services, these results can have practical significance in understanding how to achieve optimal performance.

### Improved self-regulation abilities

In this study, skills relating to normalising and allowing different types of emotions and reactions were identified. It has been found that firefighters who comply more with values such as being tough and resilient in all situations have a higher probability of PTSD [31]. Thus, the skills brought up in this study can be seen as helpful in protecting the PCIS participants from PTSD. Also, there have been indications of how emotional awareness can be related to increased resilience and coping [32]. This current study also indicates the significance of recognizing and accepting emotions. Jakubowski & Sitko-Dominik [31] found that social support was connected to the probability of PTSD in a reverse way. Our study emphasises the value of social support as a mean to recover from critical incidents that can lead to psychological burden. In addition, ‘improved interaction-based skills’ included skills to seek support for themselves. In this study, social support aspects of skills were described in different ways and actions. They were mentioned at a level where the participants have started to become active in supporting others, too. The importance of perceived social support for mental health symptoms has been revealed in a previous study by Vig et al. [33], showing that paramedics and firefighters who reported higher levels of social support were less likely to screen positive for PTSD and/or major depressive disorder. All in all, this study describes the positive consequences of improved self-regulation abilities and what these abilities are comprised of.

### New kind(s) of well-being

In general, when emergency service workers have solid skills to overcome incidents potentially resulting in psychological burden at work and their consequences, they are not overwhelmed by their experiences. Thus, they perform well in their positions and their work community, but they can also support the wider system in responding well and in a sustainable way to crisis situations. For example, the influence of the decisions paramedics make can be seen at the system level [34]. There is a clear need for a solid understanding of how emergency service workers cope with potential consequences of critical incidents but there is not very much high-quality research yet [23]. This study suggests PCIS as one potential support mechanism to help emergency service workers cope with the aftermath of critical incidents. As one interesting aspect of this study, understanding how emergency service workers form a new type of relationship with their previous burden in practice is crucial for seeing what is important to gain so that the burden does not hinder the performance, at least not too much.

### Readiness to help

In this study, skills were also manifested in ‘implementing the learned self-help skills’ that refer to, for instance, using certain techniques to support their well-being. In line with that, among emergency service workers, such as firefighters, it has been found that using mindfulness techniques, which were found to be connected to resilience, has a further connection to decreased levels of burnout [35]. In the current study, the mentioned skills and techniques were not all completely new but at least rediscovered by the participants. Interestingly, a recent study [36] following firefighters in the early years of their careers revealed that those selecting a career in emergency service have exceptional resilience against severe distress. Still, in light of the current study, coping skills can be improved. Thus, enabling education and training to develop and strengthen coping skills for a wider group of emergency service workers could benefit the whole system. This study sheds light more on understanding how readiness to help can be manifested holistically in the life of an individual emergency service worker: it can be better understood how individuals can support themselves, their clients, their colleagues and the whole work community.

## Summary

In future research, it would be beneficial to study the maintenance of these skills over longer periods than six months. Practically speaking, it would be necessary to follow up on what types of skills are most useful and effective in emergency service workers’ daily tasks and when faced with critical incidents. This would benefit also the overall education and training. In addition, the relationships between the skills and mental health symptom scores would be interesting to delve further into as well.

### Methodological considerations

The target group of the study was represented excellently (94%), which increases the trustworthiness of this study. However, with the qualitative approach, the study concentrated on self-reported and self-perceived skills that were not confirmed in other ways. In addition, asking interviewees to reflect on the past could create retrospective bias in their answers. It is also possible that they wanted to exaggerate the impacts of the seminar, for instance, because continuing to organize PCIS for emergency service workers might be desirable from their perspective. The methodology of this study does not exclude the possibility of other factors than attending PCIS affecting how participants answered in the interview. Moreover, it is not possible to ensure that all participants understood the difference between the impact of PCIS on skills and the manifestation of these skills. Also, the differentiation between these two in participants’ replies depends on the perspective and interpretation of the researcher. Thus, there is a possibility of not having completely accurate results between these two research questions. In this study, the first author who conducted the analysis is a psychologist and her theoretical understanding may affect how the results were formed in this qualitative approach. This was attempted to be reduced by reflecting on the results carefully between the two researchers.

One of the methodological constraints in this study is that the setting does not exclude the opportunity for natural healing or healing with professional or non-professional networks or support after the seminar. Since the incidents are not recent, we assume that there have been symptoms that have not disappeared during the initial natural healing that probably have been processed in the seminar and after the seminar with the contribution of it.

The original and modified PCIS are not seen as having major differences in what type of influences they may have since the modifications made for the Finnish emergency services’ purpose have not been very significant.

The study was conducted in an interdisciplinary research group, which was transparent for the participants. In relation to this, it was assumed that when knowing the educational and professional experience of the interviewer, it could also be easier for the participants to describe potentially more complex or sensitive details of their experiences regarding the research focus. However, the psychologist background of the interviewer may have influenced how she interpreted the data in the analysis phase. Based on the evaluation of the research group, the interviews resulted in saturated data with excellent depth.

Based on the results of this study, PCIS participants were a feasible target group for this kind of study aim, as many of them had already experienced apparently severe mental burden and they were willing to learn how to cope with it. This combination resulted in deep descriptions of the skills gained to overcome the psychological burden.

As the approach was qualitative, the results focused more on depth and context-specific understanding rather than broad generalisations [37]. In future studies, the experiences of PCIS participants should be compared to firefighters and paramedics who received different support interventions or none in order to confirm the results regarding PCIS impact. In addition, comparative studies involving interviews, for instance, a month later and a longer time after, would be beneficial in determining the impacts of PCIS in helping emergency service workers cope. However, the foundations this research has created and the conclusions drawn leave the door open for other researchers in the future.

## Conclusions

In this study, the participating emergency service workers reported experiencing improved performance abilities and self-regulation abilities six months after attending the PCIS. Further, the participants reported new kinds of well-being practices and a renewed readiness to help. In addition, the results emphasise the value of social support as one of the means to recover from critical incidents among the group of emergency service workers.

In the future, longer follow-up studies and different methodological approaches are needed to investigate how acquired skills from PCIS practically affect emergency service workers and their well-being.

Overall, supporting emergency service workers to cope with incidents that can lead to psychological burden at work is a crucial way of consequence risk reduction at an individual level and a system level. The emergency service workers perform well in their positions and work community with such support. They can support the wider system and society in responding well, sustainably, to crisis situations. Based on the results of this study, it is concluded that attending a PCIS may be effective in developing skills to overcome critical incidents that can lead to psychological burden.

### Electronic supplementary material

Below is the link to the electronic supplementary material.

**Supplementary Material 1:** The program of PCIS in spring 2021

**Supplementary Material 2:** Interview guide

## Data Availability

The datasets generated and analysed during the current study are not publicly available due to the inclusion of sensitive information and the extent of the informed consent provided by the participants, but are available from the corresponding author upon reasonable request.

## Abbreviations

EMD: Eye Movement Desensitization
EMDR: Eye Movement Desensitization and Reprocessing
FBI: Federal Bureau of Investigation
PCIS: Post-Critical Incident Seminar
PTSD: Post-Traumatic Stress Disorder
