# Supplementary material for: Supporting emergency service workers to cope with critical incidents that can lead to psychological burden at work - developing skills in the Post Critical Incident Seminar: a qualitative interview study
Source: BMC Psychol. 2024 Jan 22;12:44. doi: 10.1186/s40359-024-01534-x (PMC10804621; doi:10.1186/s40359-024-01534-x)
Supplement: Supplementary file 1 — Supplementary Material 1: The program of PCIS in spring 2021 [file 40359_2024_1534_MOESM1_ESM.docx]

APPENDIX 1.

**THE PROGRAM OF PCIS IN SPRING 2021**

**Tuesday – First Day**

**09.00 Opening and introductions**

- Confidentiality, openess and basic rules
- Schedules
- Post Critical Incident Seminar –model, history and general overview
- EMDR; what it is and how it works
- Introducing participants

**10.30 Instructor’s story I and II**

**12.00 Lunch**

**12.45 Instructor’s story III**

**13.15 The burdening incidents of the participants**

Every participant tells what made them apply to PCIS

The day ends around 17 with shared relaxing/breathing exercise.

**18.00 Dinner**

Collective meeting in the evening. Different shared activities. Everyone’s situation is taken into consideration.

**Wednesday – Second Day**

**08.00 Good morning and check-in. Remarks?**

How did the night go, and with what kind of thoughts did this day start?

**09.00 The recovery stages of emotional life - lecture of a professional (psychotherapist).**

**10.40 Starting small groups – Three small groups are gathering.**

**11.00 Small group discussions / Meetings with a professional (psychotherapist, not considered as health care by the PCIS organisers) (EMDR)**

One person from the group leaves to meet the professional (psychotherapist)

**12.00 Lunch**

**13.00 Outdoor activities in small groups / Meetings with a professional (psychotherapist, not considered as health care by the PCIS organisers) (EMDR)**

**14.00 The role balance of first-line responder and interactions – the director of the PCIS / Meetings with a professional (psychotherapist, not considered as health care by the PCIS organisers) (EMDR)**

**15.30 Small group discussion / Meetings with a professional (psychotherapist, not considered as health care by the PCIS organisers) (EMDR)**

**16.30 The influence of the work of front-line helper to family life – visitor lecturer, national coordinator of social and crisis emergency services / Meetings with a professional (psychotherapist, not considered as health care by the PCIS organisers) (EMDR)**

The day ends with a collective meeting when all the participants have had a meeting with a professional (psychotherapist).

Feelings and wrapping up the programme with breathing / relaxing technique

**n. 18.00 Dinner**

Collective meeting in the evening. Different shared activities. Everyone’s situation is taken into consideration.

**Thursday – Third Day**

**08.00 Good morning and check-in. Remarks?**

How did the night go? What kind of thoughts do they have about this last day?

**08.30 Preparing and recovering – detective sergeant / Meetings with a professional (psychotherapist, not considered as health care by the PCIS organisers) (EMDR)**

**10.30 Small group discussion / Second meeting with a professional (psychotherapist, not considered as health care by the PCIS organisers) (EMDR)**

**11.40 Checking out from accommodation**

**12.00 Lunch**

**13.00 Outdoor activities in small groups / Second meeting with a professional (psychotherapist, not considered as health care by the PCIS organisers) (EMDR)**

**14.15 Small group discussion about coping/survival mechanisms**

**14.45 Hope! - Priest**

**15.15 Personal feedback/assessment from the participants – survey**

**15.30 Collective course assessment, considerations and final words**

**16.30 Ending for the PCIS participants – Safe travels!**

**The program schedules are directional. Breaks are taken at suitable intervals.**
