# Supplementary material for: Supporting emergency service workers to cope with critical incidents that can lead to psychological burden at work - developing skills in the Post Critical Incident Seminar: a qualitative interview study
Source: BMC Psychol. 2024 Jan 22;12:44. doi: 10.1186/s40359-024-01534-x (PMC10804621; doi:10.1186/s40359-024-01534-x)
Supplement: Supplementary file 2 — Supplementary Material 2: Interview guide [file 40359_2024_1534_MOESM2_ESM.docx]

APPENDIX 2.

**Supporting emergency service workers to cope with critical incidents that can lead to psychological burden at work - Developing skills in the Post-Critical Incident Seminar: A qualitative interview study**

**Interview guide (the original order, please see notes below)**

PCIS=Post-Critical Incident Seminar

Information and reminders before starting the recording:

- Introduction and thank you for participating.
- Are you in a quiet place, alone, with a good internet connection?
- Information on the progression of the interview: first a few background questions, then questions divided by themes.
- Personal information will not be asked and sharing it should be avoided.
- The interview will be audio recorded.
- Interview consent will be stored.
- Any questions?

*Recording starts.*

1. Background information

- age?
- years of work experience in emergency services?
- time passed since the possible critical incident that resulted in psychological burden (if any specific)?

1. The possible impacts of the PCIS on the experience of the possible critical incident and on different areas of life and psychological state

- on experiences relating to the critical incident (which was the reason for attending the PCIS)?
- on work?
- on other aspects of life?
- on psychological (or emotional) state 6 months after the PCIS?
- Positive change or growth in your work role resulting from the critical incident experience?
  - in other aspects of your life?
- The most impactful elements of the PCIS?

1. Support or treatment received and possible gaps, before and after the PCIS for reducing psychological burden

- Before the PCIS?
  - Impact on well-being?
  - Desire and feeling of needing support or treatment before the PCIS?
  - Support from colleagues, family or friends after the critical incident?
  - Support from the operational supervisor
  - Its impact on your well-being
  - If not, would you have wished for it and what kind?
- After the PCIS?
  - Impact on well-being?
  - Wish and feeling of needing treatment after the PCIS?
  - Support from colleagues, family or friends after the critical incident?
  - Support from the operational supervisor
  - Its impact on your well-being

If not, would you have wished for it and what kind?

- Other activities and treatments compared to the PCIS?

1. Impacts and manifestation of the PCIS on the competencies and skills needed to cope with work incidents that can lead to psychological burden?

- Manifestation of competencies and skills during the 6 months since participating in the PCIS?
- Most helpful competencies and skills?
  - Reasons?
  - How were they gained?
  - Maintenance and development?

Anything to add in general?

*Note: Based on the answers, additional questions were asked during the interviews (under each theme) as the conversation flowed.*

Ending the interview:

- Possible questions & comments
- Thank you.

---

Notes:

- Before the interviews, every PCIS attendee (n=16) was contacted and asked to provide written consent for participation in the interview (only one participant did not return the written consent and did not attend the interview). Additionally, these semi-structured interview themes were sent to the participants before the interviews.
- After the third interview, the researchers decided to change the order of the original third and fourth themes based on the interviewer’s understanding of how the questions were experienced and answered.
